# Electrical Stimulation for Treatment of Dysphagia Post Head Neck Cancer: A Systematic Review and Meta-Analysis

**DOI:** 10.1055/s-0043-1761175

**Published:** 2023-09-14

**Authors:** Émille Dalbem Paim, Lica Arakawa Sugueno, Vera Beatris Martins, Virgilio Gonzales Zanella, Fabricio Edler Macagnan

**Affiliations:** 1 Speech Therapy Department, Santa Casa de Misericórdia de Porto Alegre, Porto Alegre, RS, Brazil; 2 Graduate Program in Rehabilitation Sciences, Universidade Federal de Ciências da Saúde de Porto Alegre, Porto Alegre, RS, Brazil; 3 Graduate Program in Human Communication, Faculdade de Ciências Médicas da Santa Casa de São Paulo, SP, Brazil; 4 Head and Neck Surgery Department, Hospital Santa Rita, Santa Casa de Misericórdia de Porto Alegre, Porto Alegre, RS, Brazil; 5 Physical Therapy Department, Universidade Federal de Ciências da Saúde de Porto Alegre, Porto Alegre, RS, Brazil

**Keywords:** electrical stimulation, dysphagia, head and neck neoplasms, rehabilitation

## Abstract

**Introduction** Dysphagia induced by radiotherapy in the head and neck region comprises a challenging scenario and sometimes difficult rehabilitation due to the severity of the adverse effects. Some resources such as electrical stimulation have emerged as an alternative to complement the therapeutic process, but there is still no consensus on its use.

**Objective** The purpose of the present study was to evaluate, through a meta-analysis, the effect of electrical stimulation on the rehabilitation of dysphagia generated after head and neck cancer treatment.

**Data Synthesis** Four randomized controlled trials with a total of 146 participants were included. The age of the participants was 58.37 ± 1.8 years old and there was a predominance of males. The time to start the intervention ranged from 50.96 ± 40.12 months after cancer treatment. The intervention showed great heterogeneity regarding the positioning of the electrodes, parameters, duration of the stimulus, number of sessions, and intensity. No difference was identified in the following aspects: oral transit time, hyoid elevation, penetration and/or aspiration after electrostimulation. The quality of the evidence ranged from very low to moderate and high risk of bias.

**Conclusion** In this meta-analysis, we found weak evidence for small and moderate swallowing benefits in patients after radiotherapy for head and neck cancer in short-term clinical trials.

## Introduction

Dysphagia is a highly prevalent symptom in head neck cancer (HNC). 1 It is estimated that between 31 and 79% of patients with HNC experience swallowing disorders after surgery and radiotherapy (RT), two common modalities for HNC treatment. 2 Regardless of the etiology, dysphagia causes negative impacts on respiratory and nutritional functions, leading to an increase in the rate of aspiration pneumonia, malnutrition, and dehydration problems with impairment to health and quality of life (QOL). 3

Surgery compromises swallowing due to the extension of the procedures and their reconstructions, which often leads to major structural damage and tissue loss. 4 Radiotherapy (RT), despite technology advances, causes changes in muscle configuration and muscle fibrosis, reduces sensitivity, contributes to laryngeal edema, reduces laryngeal elevation, and impairs airway protection. 5 6 7 These associated factors often cause chronic dysphagia, which makes it difficult to rehabilitate and due to the predominantly irreversible nature of these changes, many patients still have swallowing difficulties for years after the end of treatment. 8

It is also commonly observed, among other clinical signs and symptoms, hyposalivation and/or increased salivary viscosity 9 that lead to the accumulation of pharyngeal residues (due to the difficulty of cleaning) and silent aspiration (during or after swallowing). 5 6 7 8 Hyposalivation also requires attention as it impairs the preparation of the food bolus and a tendency to food residue in the pharynx, which, in addition to discomfort, impairs the QOL of patients.

Hyposalivation and radioinduced fibrosis are two major challenges in daily practice. This requires a multiple approach, with adaptation of food consistency and utensils used, rhythm and volume of each portion, airway protection maneuvers and posture change. In addition, muscle training guided by the speech therapist is indicated to reduce fibrosis, improving laryngopharyngeal strength and mobility during swallowing. 10 11

There is a large number of publications available in the literature that address interventions to prevent or rehabilitate muscle dysfunctions related to dysphagia. 12 13 14 Some studies evaluate therapeutic interventions that start before cancer treatment, others during, and some in the late phase after the end of antineoplastic treatment. 15 16 Apparently, there is no consensus on which intervention should be used as a care routine, not even on the ideal start time.

More recently, some instruments, such as neuromuscular electrical stimulation (NMES), have emerged to streamline the swallowing rehabilitation process. 16 17 18 19 One of the hypotheses that tries to explain the beneficial effects of electrical stimulation on muscle preservation involves reducing the production of TGF-β1, considered an important marker of the degree of severity of muscle fibrosis. 20 21

There is evidence that NMES may promote improvement in muscle homeostasis, including changes in the type of muscle fiber. 21 After RT, the skeletal musculature of the head and neck region tends to change its muscular configuration, with a predominance of type 1 muscle fibers. 22 Type 1 muscle fibers are responsible for slow contractions and are more resistant to fatigue; however, when it comes to swallowing, it is necessary to have a muscular balance so that, in addition to resistance, there is adequate strength and hyolaryngeal mobility. 23 In this scenario, possibly, electrostimulation would enhance muscle regeneration if associated with exercises.

Some studies show potential in the technique for recovery of salivary flow, 24 25 and for dysphagia several methodologies and protocols are suggesting a lack of consensus. 21 In addition, there is still a hesitation to apply electrical stimulation on areas where cancer treatment has been carried out. This hesitancy may be related to the hypothesis that this technique could stimulate the growth or proliferation of cancer cells after treatment. Despite that, there is no published research that validates this apprehension as a scientific fact. On the other hand, it has already been demonstrated in some studies that electrical stimulation shows no positive correlation to tumorigenesis. 26 This uncertain paradigm is delaying the rehabilitation process by not having NMES being used earlier on patients' treatment, which could possibly prevent chronic effects on swallowing. 26

This fact makes it difficult for the therapist to define whether to use (or not) this resource. Therefore, the purpose of the present study was to evaluate, through a meta-analysis, the effect of electrical stimulation on the rehabilitation of dysphagia generated after HNC treatment.

The present review follows the Preferred Reporting Items for Systematic Reviews and Meta Analyses (PRISMA) guidelines 27 and, subsequently, it was registered in the International Prospective Register of Systematic Reviews (PROSPERO), with the following identification: CRD42020200248.

## Methods

Clinical trials using NMES for rehabilitation of dysphagia in all languages were included when according to the characteristics of the participants (a): adults (≥ 18 years old) who underwent RT for the treatment of head and neck tumors, with or without surgery, with any stage or severity of dysphagia objectively diagnosed by tests such as videofluoroscopy of swallowing or endoscopic evaluation of swallowing with fiber optics, the result of these tests being concluded with reliable validated measures, such as the Penetration-Aspiration Scale (PAS). 28

Proposed interventions (b): electrical stimulation alone or electrical stimulation with concomitant exercises. There was no restriction for the electrical stimulation protocol. Intervention effectiveness assessment and outcome measures (c): intervention effectiveness was classified according to swallowing function (degree of dysphagia based on objective examinations and validated scales). All validated quantitative scores measuring swallowing function in patients with dysphagia were accepted. Study types (d): Clinical trials and quasi-clinical trials examining the effect of electrical stimulation alone or electrical stimulation performed during exercise treatment for dysphagia were included. Case reports and systematic reviews were excluded.

## Search Strategy

A comprehensive electronic search strategy of MEDLINE/PubMed, Embase, the Cochrane Central Register of Controlled Trials (CENTRAL), Scielo, and PEDro was performed from their earliest record to September, 2021. The search strategies can be seen in the Supplementary Material Appendix 1 .

**Fig. 1:**
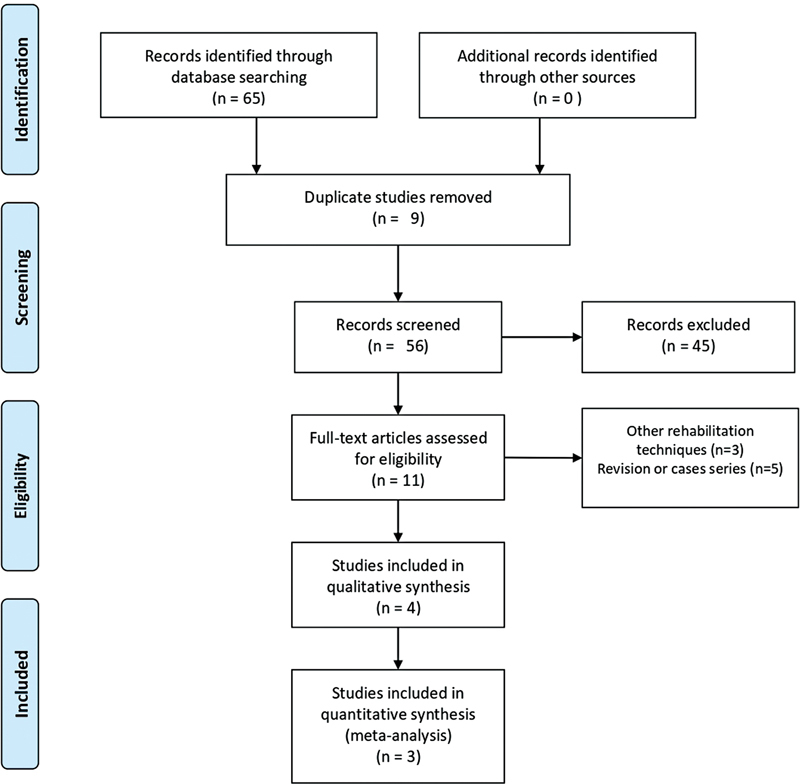
Flow chart of search and study selection.

## Study Selection

Two authors independently scanned the titles and abstracts, excluding obviously irrelevant studies. The full text of relevant articles was evaluated according to prespecified eligibility criteria. Disagreements were resolved through discussion with the corresponding author.

## Data Extraction

Two reviewers independently extracted data using a predefined data recording form and discussed disagreements by consensus. The following data were extracted: details of the study design, patient characteristics (etiology, number of patients, age, sex), intervention protocol (frequency, intensity, duration, and stimulation setting) and control group (swallowing management, exercises, sham), as well as swallowing function outcomes and assessment timing. The means and standard deviations (SDs) of change scores (change from baseline) were extracted. When data were not reported, the posttreatment mean and SD were extracted. When articles only provided the median and quartiles, the mean and SD were estimated. If important data were not available, attempts were made to contact the author by e-mail. When the authors did not respond to requests, the study was excluded.

## Statistical Analysis

The results of four studies were statistically compiled in meta-analyzes for four outcomes of interest: laryngeal elevation, laryngeal anteriorization, QOL and Penetration-Aspiration Scale (PAS). 27 Since the studies measured the same outcomes using different methods, a data compilation performed on standardized mean difference (SMD) calculates in Hedges g (M1-M2/s), where is the grouped SD; M1 is the mean of the intervention group and M2 is the mean of the control group), 29 a measure of the size of the effect in which each addition of the unit to the final result indicates that the groups differ by 1 SD. The interpretation of the findings in SMD in the present study concluded the traditional cutoff points: 0.2 represents a small effect size; 0.5 represents a medium effect size; and 0.8 represents a large effect size. 30

All analyzes were conducted using the method of inverse of variances and the DerSimonian and Laird estimator for τ2 in a random effects model, which allows to statistically incorporate the variability between studies in the final effect estimate. For continuous outcomes, data from change in relation to the baseline were preferred to deal with a more efficient analysis that confers greater statistical power when compared to the analysis of post-treatment values only. The data for each group were computed to calculate the effect size in Hedges g, presented in SMD and 95% confidence interval (CI). To include results from studies that did not report data on mean and SD in meta-analyzes, conversion of data from median to mean and interquartile range to SD was performed. In some circumstances, primary studies reported results without a measure of dispersion for values of change from baseline, such as SD or standard error. In these cases, the standard deviation was imputed from the calculation of a correlation coefficient using the SDs known from the other primary studies included in the systematic review, strictly according to Cochrane's guidance (Chapter 16.1.3.2). All analyzes were conducted using RStudio software version 1.3.1093 (R Foundation, Vienna, Austria) with the 'meta' package in R language (version 4.0.3).

## Risk of Bias

Two authors (Sugueno L. A. and Macagnan F. E.) assessed the risk of bias in individual studies, independently, using the recent modified Cochrane Collaboration's Risk of Bias assessment tool (RoB 2.0) for randomized clinical trials (RCTs). 31 ROBINS-I: a tool for assessing risk of bias in non-randomized studies of interventions (NRSIs) was used for only included NRSI 32

Each study was evaluated in relation to the following five domains: 1) bias due to the randomization process; 2) bias due to deviations from the intended interventions; 3) bias due to the lack of outcome data; 4) bias in measuring the result; 5) bias in the selection of the reported result. The risk of bias judgments were: a) low risk of bias; b) some concerns; and c) high risk of bias. If an individual domain was considered at a certain level of risk of bias, the overall risk of bias for that study was considered to be at least as severe.

Any disagreements were resolved by discussion with other three authors (Sugueno L. A., Macagnan F. E. and Zanella V. G.). An online web app robin was used to visualizing the risk of bias assessments as “traffic light” plots of the domain-level judgment for each individual result; and “weighted bar” plots of the distribution of risk-of-bias judgment within each bias domain.

## Evaluation of Heterogeneity

Statistical heterogeneity was assessed quantitatively using the I ^2^ statistic, in addition to the χ2 significance test. The interpretation of statistical heterogeneity followed Cochrane's guidelines. An I ^2^ up to 40% represents negligible heterogeneity; 30 to 60% represents moderate heterogeneity; 50 to 90% represents substantial heterogeneity; and 75 to 100% represents built-in heterogeneity.

## Evaluation of the Quality of Evidence by the Grade System

The overall quality of the evidence was assessed according to the Grading of Recommendations Assessment, Development and Evaluation (GRADE) approach. 33 For each outcome, the quality of the evidence was formed as 'high' and subsequently graded down to the 'moderate', 'low', or 'very low' quality levels, depending on the assessment of five criteria: risk of bias from individual studies, indirect indemnity, heterogeneity, imprecision, and risk of publication bias.

## Results

A total of 65 studies were yielded. Using the EndNote7 exact duplicate finder, 56 studies remained after the duplication. Forty-five studies were excluded after analyzing the title and summary. After further revision of the full text of the 11 articles, 3 involved other rehabilitation techniques (not electrical stimulation) and 5 were case series. No additional articles were included by manually searching for reference lists and citation tracking. Finally, 4 studies involving 146 patients were considered eligible for qualitative analysis. 16 17 18 19 For the meta-analysis, two studies were included for oral transit time (OTT), 16 17 two for penetration and aspiration scale (PAS), 17 18 two for hyoid anteriorization (HA), 17 18 two for hyoid elevation (HE), 17 18 and three for quality of life (QL) 17 19 as shown in Figure 1 .

## Study Data

The information extracted from the included studies is summarized in Table 1 . The articles were published between 2009 and 2016. The study included 146 participants aged 58.37 ± 1.8 years old and there was a male predominance (70%). The time to start the intervention ranged from 50.96 ± 40.12 months after cancer treatment. With the exception of one study, 21 the entire sample involved exclusive or associated radiotherapy as a treatment modality. Only one study described the RT method by intensity modulated radiotherapy (IMRT). 18

**Table 1:** Characteristics of included studies

Author, Year	*n* (total)	*n* (GI / GC)	Age (mean ± SD, years)	Male (%)	RT (%)	IMRT (%)	TPT (mean ± SD, %)	GI	GC	Analyzed Parameters
Langmore et al., 2016 18	40	20/20	61.9 ± 6.9	85,7	100	51.5	53.7 ± 60.9	FES + exercises	exercises+ SHAM*	PAS, OPSE, LE, AL, RP, QOL
Lin et al., 2011 17	20	10/10	54.2 ± 2.6	60	100	NR	9.1 ± 4.0	FES+ exercises	exercises	PAS, OTT, PTT, PDT; LE, AL, PR, QOL
Long et al., 2013 16	60	31/29	56.1 ± 0.5	48.3	100	NR	63.1 ± 6.5	FES+ exercises	exercises	WST , OTT, SRT , PTT, LCD
Ryu et al., 2009 19	26	14/12	61.4 ± 10.6	86.1	50	NR	NR	FES+ exercises	exercises+ SHAM**	FDS;CDS ASHA NOMS, MDADI

Abbreviations: AL, anteriorization laryngeal; ASHA NOMS, American speech-language-hearing association national outcome measurement system; FDS, functional dysphagia scale; GC, Control group; GI, intervention group; LCD, laryngeal closure duration; LE, laryngeal elevation; MDADI, M.D. Anderson dysphagia inventory; NR, not reported; OPSE, oropharyngeal swallow efficiency; OTT, oral transit time; PAS, penetration and aspiration scale; PDT, pharyngeal delay time ; PTT, pharyngeal transit time; QOL, quality of life; RP, residue pharyngeal; RT, radiotherapy; SD, standard deviation; SHAM, stimulation electrical* off or TENS**; SRT, swallow reaction; TPT, time post treatment; WST, water swallow test.

Regarding the region of cancer treatment, two studies involved only the nasopharynx, 16 17 and the others included in addition to the nasopharynx the oral cavity, the oropharynx, the larynx, and the hypopharynx. 18 19

## Neuromuscular Electrical Stimulation Data

The application of NMES showed high heterogeneity as to the position of the electrodes, parameters and protocols, duration of the stimulus, number of sessions, and intensity. All studies used a functional electrical stimulation (FES) current and the frequencies varied from 70 to 80 Hz, the pulse width from 300 to 700 uS, and the other parameters such as up and down ramp, time on and time off were described by only one article, 18 the others used equipment with closed programming, which did not allow changes. 16 17 18 The duration of the electrical stimulus varied from 30 19 to 60 16 17 18 minutes and the maximum intensity was reported in only one study, 16 the others reported as the maximum level of tolerance. 16 17 18 19 Adverse effects were not mentioned by any study, the other information on the application of electrical stimulation can be found in Table 2 .

**Table 2:** Characteristics of electrical stimulation

Author Year	Parameters	Position of electrodes	Duration (minutes)	FR (per week)	Equipment	Intensity (mA)
Langmore et al., 2016 18	FR 70Hz; 300Us (130-300); TON (2-4); TOFF (12-16); RO (2-4); RD 0	1 channel in the submandibular region	5 warm up + 60 with exercises	6x	BMR Neuro Tech (NT)2000	NR
Lin et al., 2011 17	80Hz , 700Us	3A/3B	60	1-3x	Vital Stim	MTI
Long et al., 2013 16	80 Hz, 700 Us	3A/3B	60	5x	Vital Stim	0-25 increasing 5 until MTI
Ryu et al., 2009 19	80 Hz, 700 Us	3A/3B	30	5x	Vital Stim	MTI

Abbreviations: FR, Frequency; MTI, maximum tolerated intensity; NR, not reported; RD, ramp down; RO, ramp On; TOFF, OFF time; TON, On Time; Us, pulse width.

## Swallowing Data

Different scales were found to assess the biomechanics of swallowing and oral intake, showing high heterogeneity. None of the selected outcomes were homogeneously assessed in all included studies.

All included studies associated exercises with the main intervention through NMES. 16 17 18 19 In the control groups, in addition to general guidelines for food, exercises traditionally used for swallowing rehabilitation were also invented. One study, 19 in addition to the exercises and NMES, used a dilation balloon.

**Fig. 2:**
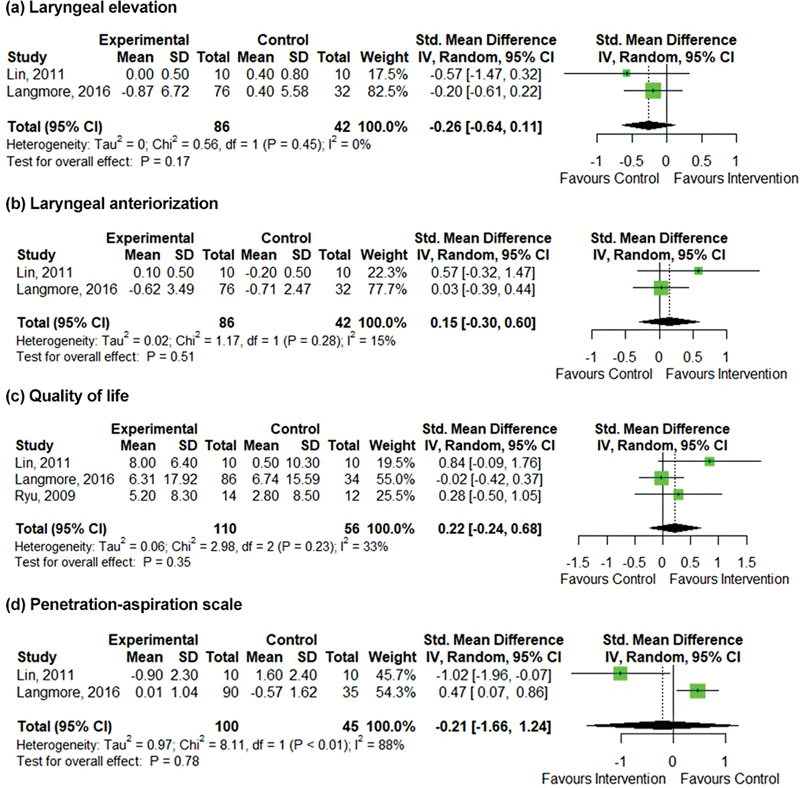
Forest plot: effects of e on Hyoid Elevation (HE) (A), Hyoid Anteriorization (HA) (B), Quality of Life (C), and Penetration Aspiration Scale (D).

Among the analyzed outcomes, studies included oral transit time (OTT) (DMP = - 1.19; 95%CI: - 3.47–1.10), 18 19 pharyngeal transit time (PTT), 18 19 pharyngeal residue (PF), 19 hyoid anteriorization (HA) (DMP = 0.15; 95%CI: - 0.30–0.60), 19 20 hyoid elevation (HE) (DMP = - 0.26; 95%CI: - 0.64–0.11), 19 20 penetration and/or aspiration (PAS) (DMP = -0.21; 95%CI: - 1.66–1.24). 19 20 The meta-analysis information for OTT, HE, HA, PAS and QOL is shown in Figure 2 .

Despite the great interest in evaluating the effect of electrical stimulation on the pharyngeal residue, it was not possible due to the variability in measurement, and it is not possible to unify it. To assess the degree of penetration and/or aspiration, the most used scale was the PAS. 27

Three studies evaluated QOL, of which one 20 used the Head and Neck Cancer Inventory (HNCI), which includes aspects related to swallowing, and two 19 20 used the MD Anderson Dysphagia Inventory (MDADI), which is specifically validated to assess the impact of dysphagia on QOL.

## Risk of Bias

**Fig. 3:**
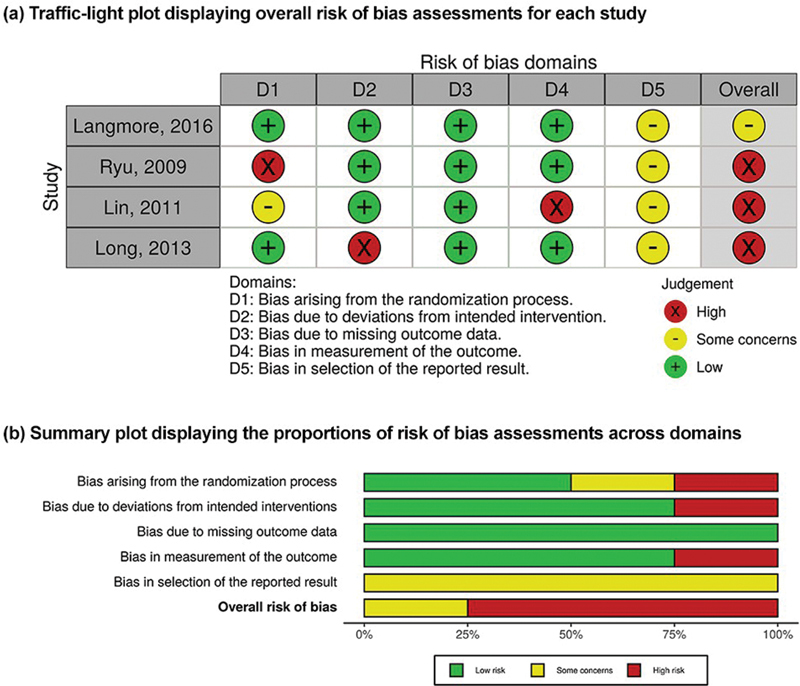
Assessment of the risk of bias (A) and risk of bias summary (B).

The risk of bias for the included studies was assessed with ROB 2.0, and the results are presented in Figure 3 . Three studies 16 17 19 were assessed as high risk of bias due to measurement of the outcome and randomization process, deviation from intended intervention, measurement of the outcome, and one had some concerns in the selection of reported results. 18

## Assessment of the Quality of Evidence

The Summary of Findings ( Table 3 ) presents an assessment of the quality of the evidence by the GRADE system, with judgments for each outcome. The quality of the evidence ranged from very low to moderate. The justifications for each judgment are assessed in greater detail in Table 3 .

**Table 3:** Summary of results: effects of electrical stimulation on different aspects of swallowing

Population: head and neck cancer patients Context: outpatient and/or hospitalized Intervention: electrical stimulation Comparison: usual care or placebo					
Outcomes a	Participants, *n* (Studies, *n* )	Compiled Studies	Statistical heterogeneity ( * I ^2^* , %)	Effect Size (DMP, 95% CI)	Quality of evidence (GRADE)
Hyoid Elevation	128 (2)	17 18	0.0%	DMP, -0.26 (-0.64 a 0.11)	⊕⊕⊕◯ ** MODERATE b**
Hyoid Anteriorization	128 (2)	17 18	15.0%	DMP, 0.15 (-0.30 a 0.60)	⊕◯◯◯ ** VERY LOW bcd**
Quality of life	166 (3)	17 18 19	33.0%	DMP, 0.22 (-0.24 a 0.68)	⊕⊕⊕◯ ** MODERATE b**
Oral transit time	80 (2)	16 17	94.0%	DMP, -1.19 (-3.47 a 1.10)	⊕⊕⊕◯ ** MODERATE b**
Penetration-aspiration scale	145 (2)	17 18	88.0%	DMP, -0.21 (-1.66 a 1.24)	⊕◯◯◯ ** VERY LOW bcd**

Abbreviations: DMP, standardized mean difference; 95% CI, 95% confidence interval.

a Means represent the post-treatment value of each group and DMP represents the standardized difference in Hedges g between groups in the post-treatment means GRADE approach to assess the quality of evidence.

b Graduated down to a level due to risk of bias in primary studies, assessed by the RoB 2 instrument.

c Graduated down to one level due to important statistical heterogeneity in the final result, not explained by subgroup analyzes or meta-regression.

d Graduated down to a level due to high inaccuracy, with a low number of participants included (< 200) and wide confidence intervals that simultaneously encompass clinically relevant benefits and harms.

## Discussion

After analyzing the data produced in the present systematic review, we found no beneficial effects of NMES treatment on swallowing rehabilitation of patients with NHC treated with RT. The results showed that, after treatment with NMES, the performance in the different swallowing tests was similar to that of the volunteers in the control group. The studies showed important discrepancies in relation to the time of completion of the cancer treatment and the beginning of the intervention and involved patients with acute and chronic effects on swallowing, making the findings variable, heterogeneous, and, sometimes, inaccurate. Among all the analyzes performed, only the laryngeal excursion demonstrated a certain benefit with electrical stimulation. However, the risk of bias was high, and the quality of evidence measured using the GRADE scale ranged from very low to moderate.

Although NMES has shown a positive effect on swallowing 16 17 19 and on QOL, 16 17 18 19 these results cannot be generalized due to the losses related to the randomization and blinding process that sometimes was not clearly described, showing heterogeneity in the design and instruments used, data analysis, reduced sample size, high number of losses and analysis without intention to treat, nonassessment of adherence and divergence in intervention protocols despite using the same NMES technique. These aspects are reflected on the grade going downwards in the analysis of the quality of the evidence through GRADE, in which there was important statistical heterogeneity in the final result and high imprecision, with a low number of participants included (< 200) and wide confidence intervals that encompass simultaneously clinically relevant benefits and harms. The previous reported difficulties were found in the study that did not identify a benefit with NMES on swallowing. 18

The parameters of the electrical stimulus used in each therapeutic program vary significantly between studies. The frequency of the electric pulse ranged from 70 18 to 80 Hz, 16 17 19 the pulse width range from 300 18 to 700 ms, 16 17 19 the total time per electrical treatment session varied from 30 to 60 minutes, and the intensity of the electric current was reported in only 1 study (maximum of 25 mA); in the other clinical trials, the intensity was progressively increased up to the maximum tolerance level. The muscular contraction produced by NMES can be controlled by the manipulation of the parameters of frequency, intensity, and duration of the impulse. There is a tendency for frequencies < 40 to 50 Hz to recruit a greater number of slow contraction fibers (type I), which are more resistant to fatigue, while higher frequencies recruit faster contraction fibers (type II) that are less resistant to fatigue. 34 35 36 37 The possibility to vary the configurations according to the desired objective is important and favors rehabilitation since the therapeutic targets change along the way according to the performance of the patient; however, only one study used open programming equipment that allows the configuration of the therapy adapting to the particularities of each individual. 18

If we analyze the composition of the suprahyoid musculature, which is the main musculature responsible for the hyolaryngeal excursion, it is possible to notice that 45.7% is constituted by type 2 fibers, 34.7% by type 1 fibers, and 19.5% by type 2X or hybrid fibers. In this concept, using programming during electrostimulation that mainly recruits type II fibers (70 Hz) as observed in the included studies 16 17 18 19 may, in fact, not promote gains in hyolaryngeal excursion or produce a limited effect size. Individualizing the protocols, reflecting on muscle composition, and varying the parameters considering the recovery of muscle homeostasis prior to radiotherapy (low, medium, and high frequencies) may be the way to recruit the musculature as a whole and promote a functional change in swallowing.

The anatomical location where the electrodes were placed was another factor that differed between studies. The supra and infrahyoid segments were stimulated in three studies, 16 17 19 while only one stimulated the suprahyoid region, 18 specifically in submandibular and mylohyoid sections. A meta-analysis analyzed the effect of NMES in different populations, in which studies involving supra- and infrahyoid stimulation demonstrated greater potential for rehabilitation of the swallowing biomechanics. 38 This is a particularly important aspect to be considered in radioinduced fibrosis, where the swallowing movement as a whole is compromised. Selective stimulation of the suprahyoid region may limit the extent of the benefit of the rehabilitation of hyolaryngeal mobility. If there is no contraindication for the placement of the electrodes in the supra- and infrahyoid muscles, electrical stimulation of both regions must be encouraged. Perhaps this is also a justification for the divergence in results between studies.

Another aspect that can influence the depth of the electrical impulse and the ability to overcome radiation-induced fibrosis is the pulse width. Most studies involving electrical stimulation for dysphagia rehabilitation are directed to patients with neurological disorders, especially after stroke. However, it is not possible to generalize the findings in these studies to the cancer population, especially after head and neck cancer and RT sequelae. This is because there are anatomical changes caused by the surgery and in the muscle configuration, which makes it difficult to create and apply a single protocol (parameters and electrode positions) for all patients.

In our clinical experience, the best results in relation to hyolaryngeal excursion and sensory changes promoted by RT occur with the development of individualized programs and with the modification of parameters and electrode positioning in the course of rehabilitation in association with exercises. The difficulty of carrying out research with patients with head and neck cancer is understood, and the need to standardize information to reduce the risk of bias but to evaluate different parameters stratifying the sample by groups after time after treatment, tumor region, age and staging seems to favor the understanding of which parameters and in which patients the relationship of the use of NMES may be more favorable. This reflection allows us to infer the reason why there is so much divergence in the literature and the difficulty in deciding as to whether or not to use NMES as an ally in the rehabilitation of dysphagia.

In all studies included in the present systematic review, electrical stimulation was associated with exercises traditionally used for swallowing rehabilitation. Therefore, one must consider the variations in the therapeutic electrical stimulation procedures, as well as the different exercise protocols employed. In addition to electrostimulation, a study used balloon dilation after all sessions of NMES. 16 There is evidence that balloon dilation promotes food transit benefits since it expands the digestive tract, whether pharyngeal or esophageal, especially in cases of stenosis. 39 The dilation balloon, in this case, is characterized as a concomitant intervention, with confusion bias, as it is not possible to analyze separately what was the effect of NMES and what was the effect of the dilation balloon on swallowing since there is evidence that the latter directly interferes with function. 38 39

In addition to the different aspects related to the electrical stimulation protocols and exercise program, the adherence to swallowing exercises plays a central role in the prognosis of dysphagia rehabilitation, but only one study clearly reported the measures used to control the involvement of the patients in the program. In the study by Lin et al., 17 adherence control was carried out every 2 weeks by means of a telephone call. In addition to checking adherence, telephone contact was also used to encourage participation and reinforce the importance of home exercises for the rehabilitation program, this monitoring possibly reflected in the large size of the effect obtained (0.91).

The time that elapses after the cancer treatment ends determines the type of side effects observed. Treating the effects of the acute phase increases the chances of obtaining better results in the rehabilitation of dysphagia. However, one of the studies included in the review did not report this information. 19 In the other studies, 16 17 18 19 there was great variability, but a larger size of the intervention effect was observed in a study 17 in which the time between the end of cancer treatment and the beginning of the intervention was shorter. It is possible to assume that the prognosis of dysphagia rehabilitation is more favorable when instituted early, because the longer the time, the greater the difficulty of management due to chronicity, especially in the case of radioinduced fibrosis. However, the limited number of articles included and the great variability found in the post-RT time do not allow this inference (50,96 ± 40,12 months).

There is evidence to show that radiotherapy affects the muscle repair mechanism, significantly reducing the number of satellite cells that are responsible in part for this regeneration. 40 41 The dose-response effect is well-established, because the greater the severity of fibrosis, the greater the limitation of the muscle response to training, 42 although the skeletal muscle has the ability to modify its structure and function in response to factors such as denervation, exercise, and electrical stimulation. 42

Exercise is one of the most effective strategies for maintaining and recovering muscle function; coupled with this, there is evidence that NMES is a method that improves performance and muscle structure as a whole. 43 The mechanism of NMES on swallowing is still unclear, but there are some theories, one of which is the possibility of the electric current promoting greater resistance to swallowing when the infrahyoid muscle is stimulated, with which the individual should make a greater effort to overcome the NMES barrier and thus improve its range of motion during the hyolaryngeal excursion. In addition, there is evidence that NMES can promote neural adaptations through afferent feedback to the spinal cord during contractions triggered by stimulation, increased isometric strength and modification of the type of muscle fiber. 43

Another aspect that has not been tested in this population is the associated use of NMES with resistance exercises. The literature has shown gains in hyolaryngeal excursion and movement speed during swallowing 43 , aspects that are altered in radio-induced dysphagia. The surveys included do not specify the exercises used and generally approach the exercises or involved Supersupraglottic, Mendelsohn, Swallows Effortful 18 , but with null results or with a reduced effect size both in the NMES group and in the group that performed only the exercises. It is believed that to overcome the scenario of radioinduced fibrosis and the impact it causes on swallowing, higher intensity training is necessary, with exercises that involve resistance and use resources as allies of therapy; in this case, NMES.

Despite the limitations identified after careful analysis, the positive findings found in the studies cannot be ruled out, 16 17 19 such as: increased speed of hyoid movement, reduced stasis in pyriform recesses, less impairment in swallowing over 3 months. It was noticed that in studies in which there was a gain in the hyolaryngeal excursion 16 17 19 (HE and HA) both in range of motion and speed, a reduction in the PAS scale was also identified. This may be associated with the importance of this mechanism in swallowing and airway protection, which impacted on the reduction of episodes of penetration and/or laryngotracheal aspiration. These functional changes, even if slight, may be related to NMES and changes in muscle structure and function that promote the reactivation of genes that were inactivated and modify the skeletal muscle phenotype 44 and the configuration of muscle fibers. Predominance of type 2A fibers and increased expression of the *MyHC2A* gene, which represents an increase in muscle strength and rapid contraction, are interdependent factors for swallowing that demand a quick and precise response and are potentiated through NMES. 38 40 41

All included studies that evaluated QOL identified a positive change after NMES, mainly in aspects related to swallowing function. 16 17 19 Only one of the studies did not identify an effect on swallowing; however, interestingly, in the assessment of QOL, patients reported improvement mainly in aspects related to the speed of eating and were already able to eat food in public without discomfort. 18 Perhaps the sensitivity of the instruments used to measure such outcomes was not sufficient to detect changes in the swallowing function, since it is unlikely that, if the individuals followed the exercise protocol (twice a day, 6 days a week), there have not been changes over the course of 3 months.

However, new studies should be encouraged, because, with larger samples and with greater methodological rigor, it is possible that the results lead to a favorable indication of the use of NMES for the rehabilitation of dysphagia in this population. Finally, it is essential that the assessment of dysphagia be more uniform, that the protocol of the swallowing rehabilitation exercise program must be better described and also more standardized. The anatomical location of the electrode attachment points should consider the wider involvement of the swallowing muscles and the parameters of the electric stimulation therapy need to be better described, especially in relation to the intensity of the electric current.

## Final Comments

In the present meta-analysis, we found weak evidence for small and moderate swallowing benefits in patients after RT for HNC in short-term clinical trials. Due to the limited quality of the evidence, our findings require further confirmation with robust randomized controlled trials.
