# Supplementary material for: Electrical Stimulation for Treatment of Dysphagia Post Head Neck Cancer: A Systematic Review and Meta-Analysis
Source: Int Arch Otorhinolaryngol. 2023 Sep 14;28(2):e339–49. doi: 10.1055/s-0043-1761175 (PMC11008950; doi:10.1055/s-0043-1761175)
Supplement: Supplementary file 1 — Supplementary Material [file 10-1055-s-0043-1761175-s221307.pdf]

| | | |
|---|---|---|
| **PUBMED SEARCH STRATEGY** | | |
| #1 | "head and neck neoplasms" [MeSH Terms] OR "head and neck neoplasms" [Text Word] OR "Upper Aerodigestive Tract Neoplasms" OR "Head Neoplasms" OR "Neck Neoplasms" OR "Cancer of Head" OR "Head Cancer" OR "Cancer of the Head" OR "Cancer of the Neck" | |
| #2 | "transcutaneous electric nerve stimulation" [MeSH Terms] OR TENS [Text Word] OR Electrotherapy OR "Electric Stimulation" OR "Transcutaneous Electric Nerve Stimulation" OR "Percutaneous Electrical Neuromodulations" | |
| #3 | (randomized controlled trial[Publication Type] OR (randomized[Title/Abstract] AND controlled[Title/Abstract] AND trial[Title/Abstract]) | |
| #4 | #1 AND #2 AND #3 | |
| #1 | "head and neck neoplasms" [MeSH Terms] OR "head and neck neoplasms" [Text Word] OR "Upper Aerodigestive Tract Neoplasms" OR "Head Neoplasms" OR "Neck Neoplasms" OR "Cancer of Head" OR "Head Cancer" OR "Cancer of the Head" OR "Cancer of the Neck" | |
| #2 | "transcutaneous electric nerve stimulation" [MeSH Terms] OR TENS [Text Word] OR Electrotherapy OR "Electric Stimulation" OR "Transcutaneous Electric Nerve Stimulation" OR "Percutaneous Electrical Neuromodulations" | |
| #3 | (randomized controlled trial[Publication Type] OR (randomized[Title/Abstract] AND controlled[Title/Abstract] AND trial[Title/Abstract]) | |
| #4 | #1 AND #2 AND #3 | |
| **SCIELO SEARCH STRATEGY** | | |
| #1 | head and neck neoplasms AND transcutaneous electric nerve stimulation AND randomized clinical trial | |
| **EMBASE SEARCH STRATEGY** | | |
| #1 | 'head and neck tumor'/exp | |
| #2 | 'head neoplasms'/exp | |
| #3 | ('neck neoplasms'/exp | |
| #4 | 'functional assessment of cancer therapy head and neck'/exp | |
| #5 | 'transcutaneous electrical nerve stimulation'/exp | |
| #6 | 'electrotherapy'/exp | |
| #7 | 'electrostimulation'/exp | |
| #8 | 'crossover procedure':de OR 'double-blind procedure':de OR 'randomized controlled trial':de OR 'single-blind procedure':de OR random*:de,ab,ti OR factorial*:de,ab,ti OR crossover*:de,ab,ti OR ((cross NEXT/1 over*):de,ab,ti) OR placebo*:de,ab,ti OR ((doubl* NEAR/1 blind*):de,ab,ti) OR ((singl* NEAR/1 blind*):de,ab,ti) OR assign*:de,ab,ti OR allocat*:de,ab,ti OR volunteer*:de,ab,ti | |
| #9 | #1 OR #2 OR #3 OR #4 | |
| #10 | #5 OR #6 OR #7 | |
| #11 | #8 AND #9 AND #10 | |
| **THE COCHRANE CENTRAL REGISTER OF CONTROLLED TRIALS (CENTRAL) SEARCH STRATEGY** | | |
| #1 | MeSH descriptor: [Head and Neck Neoplasms] explode all trees | |
| #2 | MeSH descriptor: [Transcutaneous Electric Nerve Stimulation] explode all trees | |
| #3 | MeSH descriptor: [Electric Stimulation Therapy] explode all trees | |
| #4 | MeSH descriptor: [Electric Stimulation] explode all trees | |
| #5 | #2 OR #3 OR #4 | |
| #6 | #1 AND #5 | |
| **PEDRO SEARCH STRATEGY (ADVANCED SEARCH)** | | |
| Abstract & Title | dysphagia | Swallowing |
| Therapy | electrotherapies, heat, cold | electrotherapies, heat, cold |
| Problem | no selection | no selection |
| Body part | head or neck | head or neck |
| Subdiscipline | no selection | no selection |
| Topic | no selection | no selection |
| Method | clinical trial | clinical trial |
| Match all search terms | AND | AND |

**Supplementary Material Appendix 1** Search strategies.

 © 2023. Fundação Otorrinolaringologia. All rights reserved.
